# Supplementary material for: Content-rich biological network constructed by mining PubMed abstracts
Source: BMC Bioinformatics. 2004 Oct 8;5:147. doi: 10.1186/1471-2105-5-147 (PMC528731; doi:10.1186/1471-2105-5-147)
Supplement: Additional File 2 — The original results of the above study (non-essential files are deleted to keep the file size under the limit set by BMC bioinformatics). [file 1471-2105-5-147-S2.bz2 › chilibotAdditionalFile2/dip10/137ID7816143E492/html/FOS_JUN.html]

 


 **FOS** and **JUN** 
  
Found 4259 abstracts in PubMed, retrieved 10.  
 

 What does Google say? 
 PDF only 
| .edu only 

---

**Interactive relationship** (e.g. stimulation, inhibition, etc)

**Inhibitory relationship**- Gene expression for  **Fos**  was suppressed at both times, while that for Fosl1 and  **Jun**  was augmented at 12 h and suppressed at 48 h.  Ref: 12875578 Acta Otolaryngol, 2003
**Neutral relationship**

**Non-interactive relationship** (e.g. studied together, co-existance, homology, etc.)

- Oncogenes, such as c myc, c  **fos** , and c  **jun** , are also up regulated in this model.  Ref: 12855652 Clin Cancer Res, 2003
- Western blots for AP 1  [ **JUN** ]  and its signal mediators Erk and JNK showed that expression of  **Fos**  and JNK were decreased by the addition of FZFAT at 300 microg ml.  Ref: 12856862 Am J Chin Med, 2003
- CHOP dimerizes with and inhibits the binding of C EBP related transcription factors to their consensus DNA target sequences and also forms novel complexes with other transcriptional proteins e.g. c  **jun** , c  **fos** .  Ref: 12876286 J Biol ChemJ Biol Chem, 2003
- Supershift electrophoretic mobility shift assay indicated that JunB, JunD, and c  **Fos**  components of AP 1  [ **JUN** ]  were particularly affected.  Ref: 12857751 J Biol ChemJ Biol Chem, 2003
- In addition, using an in vitro model of ovarian cancer, we demonstrated that malignant transformation of ROSE cells resulted in alteration of downstream effectors of the EGFR pathway, as exemplified by aberrant expression of p66Shc, c  **Jun** , c Myc, c  **Fos** , Lot1, p21Cip Waf, and cdc25A.  Ref: 12849999 Biochem Biophys Res Commun, 2003
- Both VPA and lithium altered the expression of the early inducible genes for c  **fos**  and c  **jun**  thus promoting the expression of specific proteins.  Ref: 12890311 Int J Neuropsychopharmacol, 2003
- The inhibition of S phase induction was associated with down regulation of c Myc, but not of c  **Fos**  or c  **Jun** .  Ref: 12883011 Proc Natl Acad Sci U S AProc Natl Acad Sci U S A, 2003
- In these mice context dependent memory retrieval evoked a marked induction of c  **Fos**  and JunB, but not of c  **Jun**  and JunD, in pyramidal CA1 neurons of the dorsal hippocampus.  Ref: 12882314 Genes Brain Behav, 2003
- MATERIAL AND METHODS From cDNA microarray screening of those specimens, several gene clusters, including Nos2 and the transcription factors  **Fos** , Fosl1,  **Jun**  and Nfkb1, were identified as possible upstream regulators of Na transport protein expression.  Ref: 12875578 Acta Otolaryngol, 2003
- These results demonstrate that contextual memory retrieval is associated with de novo synthesis of specific members of the  **Fos**   **Jun**  transcription factor family.  Ref: 12882314 Genes Brain Behav, 2003
- Using specific polyclonal antisera against c  **Fos** , JunB, c  **Jun**  and JunD, we tried to identify the candidate transcription factors of the immediate early gene family which may contribute to the molecular processes during contextual memory reconsolidation.  Ref: 12882314 Genes Brain Behav, 2003
